# Injudicious use of laboratory facilities in tertiary care hospitals at rawalpindi, pakistan: a cross-sectional descriptive study

**DOI:** 10.1186/1472-6963-13-495

**Published:** 2013-11-26

**Authors:** Muhammad Farooq Malik, Dilshad Ahmed Khan, Wafa Munir Ansari, Farooq Ahmad Khan

**Affiliations:** 1 Army Medical College, National University of Science and Technology (NUST), Islamabad, Pakistan; 2 Chemical Pathology, Army Medical College, National University of Science and Technology (NUST), Islamabad, Pakistan; 3 Department of Chemical Pathology, Army Medical College, National University of Science and Technology (NUST), Islamabad, Pakistan

**Keywords:** Injudicious use, Laboratory facilities, Result misinterpretation, Health care costs

## Abstract

**Background:**

In recent years inappropriate and excessive use of clinical laboratory facilities has become a cause of concern and has led to concurrent rise in the laboratory errors and the health care costs. The aim of the study was to find out the frequency of incomplete laboratory request forms, inappropriate test requests at various professional levels and the financial impact of uncollected reports at Armed Forces Institute of Pathology (AFIP) and Combined Military Hospital (CMH) Laboratory Rawalpindi.

**Methods:**

The cross-sectional descriptive study was conducted during a three month period from April to June 2012 at AFIP and CMH Laboratory Rawalpindi. A total of 1000 laboratory request forms were collected and scrutinized for completion from AFIP (n=500) and CMH Rawalpindi laboratory (n=500). 536 request forms of costly/specialized tests from different departments of AFIP were studied to find out the professional level of test request. The total number of tests performed at AFIP during the study period and number of uncollected reports were noted. The financial impact of these uncollected reports was also calculated. Collection of data and sorting were done manually. Patient confidentiality was maintained. Microsoft excel software and SPSS-17 were used for analysis. The study was approved by the Institutional Ethical Review Committee.

**Results:**

Out of a total of 1000 forms studied none was completely filled with clinical notes being present in only 2.4% and 13% of forms sent to CMH and AFIP respectively. 62% of the expensive investigations were requested by specialists while 38% were ordered by residents and general practitioners but the percentage of avoidable expensive tests ordered by the general practitioners and residents was significantly higher than the specialists(p<0.001). A total of 9026 (40%) and 5046 (22%) diagnostic test reports were not collected from the Chemical pathology and Hematology departments respectively. Financial impact of uncollected reports from all the departments at AFIP collectively amounted to Pakistani Rupees (PKR) 3338201.

**Conclusion:**

Processing incomplete laboratory request forms and injudicious use of laboratory facilities leads to incorrect interpretation of laboratory test results affecting outcome of the overall treatment.

## Background

Clinical laboratory investigations have prime importance in diagnosis and treatment of patients. However, in recent years inappropriate and excessive use of clinical laboratory facilities has become a cause of concern. A 9.30% per annum increase in the generation of laboratory test results and a 20.2% increase in laboratory expenditures has been reported in a large tertiary care hospital in Sweden [1]. The provincial health budgets in South Africa are exceeding their allowable limits due to excessive laboratory tests requested by the clinicians [2]. This increase does not necessarily have a diagnostic and therapeutic value [3]. Excess utilization or injudicious use of laboratory facilities occurs when irrelevant or repeat laboratory testing is ordered, or when test results do not contribute towards the effective management of the patient.

Increased work load on the laboratory personnel is worsened by incomplete laboratory forms provided by the clinicians leading to rise in the rate of pre-analytical errors. Study reports that incorrect or incomplete data provided to the laboratory could significantly affect the quality and outcome of overall treatment [4]. Study states that pre-analytical errors in completing request forms may lead to incorrect interpretation and poor patient diagnosis and treatment [5]. Another major factor is lack of adequate training in ordering laboratory tests by the physicians and their use for non-targeted and vague therapeutic purposes [6].

Collection of laboratory results and their delivery to the requesting physician is also an essential phase of the clinical laboratory testing process. Results that never reach the physician affect the quality of patient care and unnecessarily waste financial health resources. A study reports a significant amount of the laboratory budget is wasted on laboratory test reports that are never collected from the laboratory [7].

Keeping the above mentioned facts in view the aim of this study was to find out the frequency of incomplete laboratory request forms, inappropriate test requests at various professional levels and uncollected reports at Armed Forces Institute of Pathology (AFIP) and Combined Military Hospital (CMH) Laboratory Rawalpindi.

Due to dire paucity of such studies in Pakistan this study has been planned to analyze the misuse of laboratory facilities so that we can access and analyze the current practices of laboratory test requests sent by physicians and improve it in future.

## Methods

### Setting and study design

The cross-sectional descriptive study was carried out over a three month period from April to June 2012 at Armed Forces Institute of Pathology and Combined Military Hospital (CMH) Laboratory, Rawalpindi, after taking approval from the Institutional Ethical Review Committee.

### Data collection and analysis

Permission to access patient files was obtained, and data were collected with the assistance of staff working in the patient records department. The information was recorded on the data analysis sheet. Patient confidentiality was maintained, names and hospital numbers were not captured on the data sheet. The major departments targeted were Chemical pathology, Hematology, Microbiology, Virology, Endocrinology and Histopathology. 1000 laboratory request forms, 500 each from AFIP and CMH Rawalpindi laboratory were selected randomly from amongst the request forms sent to all the departments. We tried to select an equal number of forms from each department to avoid any bias and for adequate representation of all the departments. The forms were scrutinized for the complete entry of patient, clinician and sample information. Both in and out patient tests were included in the study. Emergency tests were excluded. 536 request forms of costly/specialized tests sent to different departments of AFIP were scrutinized to find out the professional expertise of the requesting physician. Trainees’ acquaintance with investigation costs was assessed via a multiple‒choice questionnaire. Total number of tests performed at AFIP and CMH Rawalpindi laboratory during the study period and number of uncollected reports were noted. Laboratory reports were considered uncollected if not collected either by hand, on the telephone or via the internet within 3 months after being issued [7]. Moreover, we interviewed the physicians and pathologists at CMH Rawalpindi, MH Rawalpindi and AFIP to get their point of view regarding incomplete laboratory request forms, inappropriate test requests at various professional levels and uncollected reports all the above mentioned issues.

Detailed department-specific financial impact of the uncollected reports was also calculated by getting information from the accounts office. Cost per test was taken as only the direct cost of the test. The expensive tests were further divided into avoidable and unavoidable expensive tests as per the criteria set by Miyakis et al., [8]. Out of 1000 test request forms 150 sequential requests for histopathological examination and 100 tumor marker requests were included to be studied in detail.Requests for cytological examination were excluded. Each request form for histopathological examination was assessed for the presence and completeness of information regarding; patient’s demographic data (age, gender), clinical history or differential diagnosis, site of biopsy, description of specimen, name of referring clinician or contact number and signature. The specimen with each request form was also evaluated for quality control parameters namely size of the container relative to the specimen size, volume of the fixative at least three times the size of the specimen and identification label on the container mentioning patient’s name and or specimen. The request forms for serum α-fetoprotein (AFP), CA125, CA15-3, CA19-9, Carcinoembryonic antigen (CEA) and prostate-specific antigen (PSA) were mainly included in the study to assess the appropriateness and trend of completing tumor marker request forms. Each request form for tumor marker analysis was evaluated for the presence of information regarding patient’s age and gender, name of requesting physician, nature of sample/specimen, date of collection of sample and certain quality markers like volume of sample, appropriateness of test request and whether the tests were ordered for screening and diagnostic purposes.

Statistical analysis was performed using SPSS-17. Descriptive categorical data were presented as numbers (counts) and percentages. A frequency distribution table and bar charts were created to summarize the data.

## Results

The results regarding completion of test requisition forms are summarized in Table 1. Out of a total of 1000 forms from CMH Rawalpindi laboratory and AFIP, none was completely filled. Though name was written on 100% forms, diagnosis was written on 9% and 22% forms while clinical notes were written on 2.4% and 12.6% for CMH Laboratory and AFIP respectively. Nature of the specimen was also not entered on 39% and 41% of the forms from CMH and AFIP respectively. When physicians were asked about the importance of writing diagnosis and clinical notes on the laboratory request forms, more than 55% agreed that it is necessary in all cases while 45% said that it is necessary but not in every case. 73% of the physicians claimed that they wrote clinical notes most of the time while 18% negated writing clinical notes. When inquired about the reasons for not writing the clinical notes and diagnosis, more than 63% said it is due to the increased workload. The pathologists were of the view that diagnosis and clinical notes help in interpretation of results and should definitely be mentioned.

**Table 1:** Completion of laboratory request forms (n = 1000)

**Category**	**CMH Rawalpindi**	**AFIP**
	**n (%)**	**n (%)**
**Patient information**		
Name	500 (100%)	500 (100%)
Age	240 (48%)	360 (72%)
Gender	276 (55%)	356 (71%)
Ward/department	270 (54%)	280 (56%)
**Specimen information**		
Nature of specimen	195 (39%)	207 (41%)
Date of collection	24 (5%)	86 (17%)
**Clinical notes**		
Provisional diagnosis	45 (9%)	110 (22%)
Clinical notes	12 (2%)	63 (13%)
Physician name	426 (85%)	407 (81%)

n = Total number of laboratory request forms reviewed, % = percentage, CMH = Combined Military Hospital, AFIP = Armed Forces Institute of Pathology.

**Figure 1:**
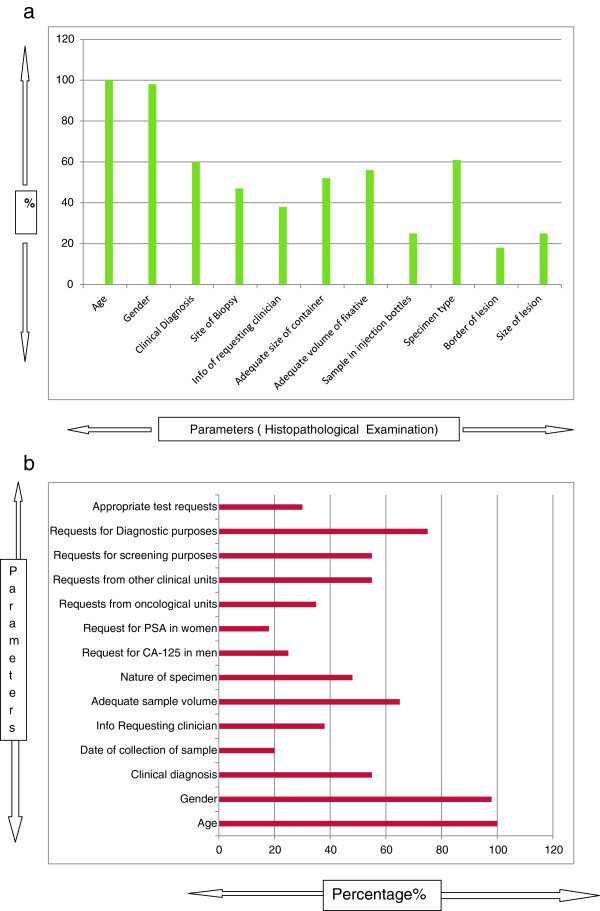
Appropriateness of sample and completion of various test request forms sent for (a) Histopathological Examination(n=150) and (b) serum tumor marker estimation.

**Figure 2:**
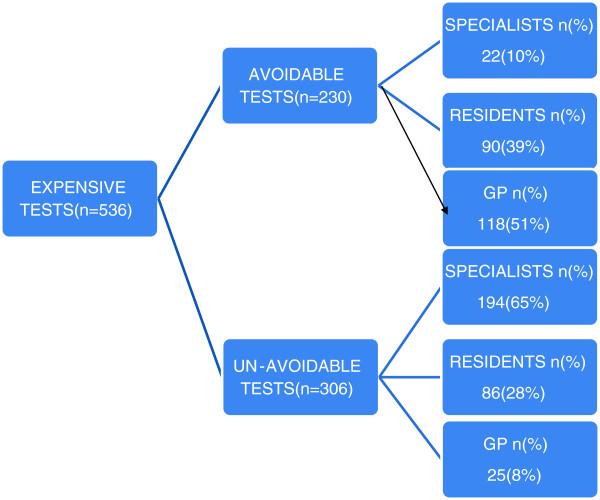
Distribution of the subgroups of expensive tests (n=536) according to the professional level of the requesting physicians.

Out of all the expensive investigations 62% were requested by specialists while remaining 38% were requested by residents and general practitioners. However, the number of avoidable expensive tests ordered by the GP and residents were significantly higher than those ordered by the specialists (Figure 1). In spite of clear instructions to restrict the advice of Vit-D to consultants only, 17% were still advised by residents and general practitioners (GP). Highest number of requests by GP and residents (69%) was seen in case of QuantiFERON-TB Gold (Table 2). Regarding their opinion about the level of request in case of expensive laboratory investigations, more than 70% agree that these expensive tests should be advised by classified specialists only. We specifically asked the referring doctors about the reason or justification of their test requests especially the expensive tests. 70% of the referring doctors had no justification and agreed that they should have consulted the specialists before advising the tests. They further suggested that to avoid this in future a three month training in the Pathology Department should be made compulsory for young doctors during their house job so that they may be made aware of the necessary protocols for advising the laboratory tests. Figure 1a, b show a detailed analysis of the appropriateness of sample and completion of test request forms for histopathological examination (n = 150) and Serum tumor marker estimation (n = 100). Figure 2, describes the distribution of the subgroups of expensive tests (n = 536) according to the professional level of the requesting physicians.

**Table 2:** Distribution of Test requests based on professional level (n = 536)

**Test**	**Specialists n (%)**	**Trainee/resident n (%)**	**GP n (%)**
FNA (n = 76)	34(45%)	22(29%)	20(26%)
Anti-CCP (n = 81)	50(62%)	19(23%)	12(15%)
PCR-HCV (n = 116)	94(81%)	16(14%)	6(5%)
VIT D (n = 100)	83(83%)	9(9%)	8(8%)
QuantiFERON-TB Gold (n = 95)	26(27%)	18(19%)	51(54%)
Coagulation profile (n = 68)	44(65%)	14(21%)	10(15%)
**Total n = 536**	**331(62%)**	**98(18%)**	**107(20%)**

n = total number of test requests sent, % = percentage, GP = General Practitioner, FNA = Fine Needle Aspiration, CCP = Cyclic Citrullinated Peptide, PCR-HCV = Polymerase Chain Reaction-Hepatitis C Virus, Vit-D = Vitamin D, TB = Tuberculosis.

A total of 180050 tests were performed at AFIP out of which 22454 reports were not collected which makes about 13% of the total reports. Highest number of uncollected reports was from Chemical pathology followed by Hematology, Virology, Microbiology, Endocrinology, Immunology and Clinical pathology. Results are summarized in Table 3. The department specific breakup of the cost of analyses for uncollected reports during the representative period are shown in Table 4. When inquired about the uncollected reports more than 91% of the physicians said that they always ensure to check the results of tests they had requested and 55% of the physicians agreed that lab tests support the establishment of diagnosis. An important finding was that more than 72% physicians agreed that number of laboratory tests can be reduced without compromising the patient care.

**Table 3:** Department-specific distribution of uncollected reports-AFIP (n = 180050)

**Department**	**Total tests performed**	**Uncollected test reports**	**Percentage**
Chemical pathology	22565	9026	40%
Hematology	22936	5046	22%
Virology	22308	2677	12%
Microbiology	23550	1884	8%
Endocrinology	24233	1454	6%
Immunology	23550	942	4%
Clinical pathology	19775	791	4%
Histopathology	21133	634	3%
**Total**	**180050**	**22454**	**13%**

n = Total number of tests performed, AFIP = Armed Forces Institute of Pathology.

**Table 4:** Department-specific financial impact of uncollected reports (Apr- June 2012) at AFIP

**Department**	**Cost (PKR)**
Endocrinology	265610
Clinical pathology	24471
Microbiology	400800
Chemical pathology	526016
Hematology	326763
Histopathology	283333
Toxicology	41316
Tumor marker	363583
Virology	933033
Immunology	173283
**Total**	**3338201 (approx 3 M)**

PKR = Pakistani Rupees, AFIP = Armed Forces Institute of Pathology, M = Million.

## Discussion

The study revealed that overall standard of completion of laboratory forms was highly objectionable. Only the name of the patient appeared on all the forms evaluated. This result is similar to that obtained by studies which showed that the patient’s name was stated on all the request forms they evaluated [9,10]. This high percentage may be owing to the fact that if the name of the patient is missing on the request form it is returned to the concerned department and the request is not processed further. Age and gender were missing on 48% and 55% of the forms received at CMH laboratory while on 72% and 71% of the forms received at AFIP. This is much higher than the figures of 5.8% and 14% obtained from a study done in Pakistan previously [11]. Age and gender are extremely important considering that the reference ranges for a number of analytes are age and sex dependent [12]. One of the reasons for not entering the age of the patient may be the fact that a number of patients coming from rural areas of Pakistan are unaware of their actual age and do not provide the correct information. Clinical notes were also sparsely entered on the forms. Correct interpretation of result may depend upon provisional diagnosis written on request form [10]. All these factors pose difficulty to the pathologist when clinically correlating the biochemical findings of the patient. Inadequate information regarding the nature of the specimen can result in use of inappropriate diagnostic technique for analyzing the biochemical parameter leading to misinterpretation of test results [13].

Another significant finding was that in spite of clear instructions on the subject more than 39% costly tests were requested by residents and general practitioners. It is obvious that clinical judgment of a resident or general practitioner may not be similar to that of a specialist who can utilize laboratory investigations more rationally using his superior clinical acumen. A study reported that excessive diagnostic test requests might be the result of inexperience or lack of knowledge about the appropriate use of tests. The major issue with non-specialists sending the test requests is that they perform defensive testing rather than targeted testing for the fear of missing out the diagnosis [14]. A study shows that out of a total of 24482 laboratory tests in an academic internal medicine department, 67.9% did not contribute at all towards management of patients [8]. Another cause of concern here is that most of the residents and general practitioners are unaware of the cost of the tests whereby it has been reported that the percentage of avoidable tests requested by the junior residents or trainees was higher than that of the senior residents [8]. A rare reason for ordering unnecessary expensive investigations may be the personal financial interest of the requesting physician which is not only unethical but should undergo strict audit procedures. Study shows that physicians who had an investment interest in an off-site clinical laboratory advised excessive and unnecessary diagnostic services to the patients [6]. An audit cycle performed on test request forms in a primary care setting decreased the number of test requests and improved the trend of ordering investigations among the general practitioners [15]. Histopathological examination and serum tumor marker request forms are the ones which especially contain scanty information. Most of the serum tumor markers are ordered inappropriately and do not conform to the International guidelines [16]. A study states that 68% of the request forms carried inappropriate test requests for serum tumor markers [17]. Tumor markers aid more in monitoring the cancer patients and have limited use in the diagnosis [18] whereas our study shows that most physicians order these tests as diagnostic aids. Another study carried out in Greece shows that there is considerable in appropriate utilization of tumor marker tests which increases the financial costs considerably [19].

Our study also demonstrated that a significant number of tests were uncollected from AFIP Rawalpindi laboratory. This unnecessary expense not only increases the workload of already overburdened laboratory staff but also prevents the introduction of newer and more sensitive/specific tests due to loss of a considerable amount of the laboratory budget on useless investigations. In a similar study it was reported that out of a total of 22445 laboratory tests, 464 test reports were not collected with 30% of the average monthly budget been wasted [7]. The physicians are in the habit of ordering a panel of tests for a particular condition with only one or two investigations out of them contributing towards clinical use. Therefore rest of the tests are never collected. Another reason may be that those patients who are entitled to free healthcare services make sure that their entire test profile is done and then never bother to collect the reports as they do not have to pay for them. Study states that federally funded health care programs should only pay for tests that are necessary for the patient and which contribute towards his medical management [20].

The strength of our study is that it is the first study of its kind to be conducted in Pakistan which has presented a complete and broad aspect of injudicious use of laboratory facilities in a reference laboratory (AFIP) of the country catering for all the different departments of pathology individually. Previous studies on this subject done in Pakistan have mainly focused their attention on the Histopathology section only. Secondly it gives a detailed overview of the true financial impact of the test reports which are never collected. A potential limitation of the study may be that it was carried out in two major laboratories in Rawalpindi which cater for tests from a number of hospitals in Punjab and Northern Pakistan however it is not representative of the test ordering and collection trend in the reference laboratories of Southern Pakistan. Moreover, other causes of pre-analytical and post-analytical errors in our laboratories other than incomplete test requisition forms and uncollected reports should also be studied in detail specially keeping in view the outcome of these errors on the management of the patients.

## Conclusions

In conclusion we recommend that that there should be adequate communication between laboratory personnel and clinicians. Medical students and house officers should be adequately exposed to the medical laboratory and should be briefed about the logical approach pertaining to test ordering and should be informed about the hazards and financial burden of excessive unnecessary test requests. As a strict check patient’s samples accompanied by incompletely filled request forms should be rejected since it may lead to inappropriate diagnosis. Lastly computerized physician order entry forms along with electronic delivery systems of laboratory reports to the requesting physician might help reduce the number of uncollected reports [21,22]. Future studies may aim to incorporate these changes into the current system and evaluate their effect on the test ordering strategies of the physicians in tertiary care centres.

## Competing interests

The authors declare that they have no competing interests.

## Authors’ contributions

DAK was involved in data acquisition, performed the data analysis and contributed towards data interpretation.MFM performed data collection and analysis and contributed towards the interpretation.WMA contributed to conception, design and acquisition of data and was involved in drafting the manuscript and revising it critically for important intellectual content. FAK performed data analysis and interpretation and gave the final approval of the version to be published. All authors read and approved the final manuscript.

## Pre-publication history

The pre-publication history for this paper can be accessed here:

http://www.biomedcentral.com/1472-6963/13/495/prepub
